# Home-based exercise interventions incorporating cardiac rehabilitation principles for people with cancer: a systematic review and meta-analysis

**DOI:** 10.1080/07853890.2026.2726615

**Published:** 2026-09-29

**Authors:** Petr Winnige, Filip Dosbaba, Martin Hartman, Garyfallia Pepera, Varsamo Antoniou, Eleni Karagianni, Francesco Perone, Rose Lin, Jing Jing Su, Ladislav Batalik

**Affiliations:** a Department of Physiotherapy, Faculty of Medicine, Masaryk University, Brno, Czech Republic; b Department of Rehabilitation, University Hospital Brno, Brno, Czech Republic; c Rehabilitation Clinic, Faculty of Medicine, Masaryk University, Brno, Czech Republic; d Clinical Exercise Physiology and Rehabilitation Research Laboratory, Department of Physiotherapy, University of Thessaly, Lamia, Greece; e Cardiac Rehabilitation Unit, Rehabilitation Clinic “Villa delle Magnolie”, Castel Morrone, Caserta, Italy; f Elaine Hubbard Center for Nursing Research on Aging, School of Nursing, University of Rochester Medical Center, New York, NY, USA; g School of Nursing, Tung Wah College, Hong Kong, China; h Department of Public Health, Faculty of Medicine, Masaryk University, Brno, Czech Republic

**Keywords:** Home-based rehabilitation, cancer, exercise-based cardiac rehabilitation, cardio-oncology, quality of life, *V*O_2_ peak, adherence, fatigue, resistance training

## Abstract

**Background:**

People with cancer are at increased cardiovascular risk due to treatment-related cardiotoxicity and shared risk factors. Exercise-based cardiac rehabilitation (exCR) may mitigate these risks, but access to center-based programs is limited. This systematic review and meta-analysis evaluated the effectiveness, safety, and adherence of home-based exCR in oncology populations.

**Methods:**

Six databases were searched in January 2025 for randomized controlled trials (RCTs) of home-based exCR in adults with cancer. Eligible interventions included structured aerobic and/or resistance exercise consistent with established CR guidelines. Primary outcomes were cardiorespiratory fitness (CRF), health-related quality of life (HRQoL), adherence, and safety. Random-effects meta-analyses used standardized mean differences (SMDs).

**Results:**

Nine RCTs (632 participants) were included. Home-based exCR improved CRF (SMD 0.42; 95% CI 0.09–0.75; *I*²=62%) and HRQoL (SMD 0.63; 95% CI 0.05–1.22; *I*²=84%). TESTEX scores indicated generally good methodological and reporting quality (mean 10/15; range 7–13). Adherence exceeded 75% in most studies reporting this outcome, and dropout was 15.2%. Adverse-event reporting was inconsistent; no clear safety signal emerged, but evidence was insufficient for firm conclusions. Strength and fatigue findings were inconsistent.

**Conclusions:**

Home-based exCR appears feasible and may improve CRF and HRQoL in people with cancer, offering an accessible alternative or complement to center-based rehabilitation. Incomplete adverse-event reporting limits safety conclusions. Future studies should evaluate full, multi-component CR models in oncology.

## Introduction

Cardiovascular disease (CVD) is a major cause of morbidity and mortality among people diagnosed with cancer, largely due to the direct cardiotoxic effects of cancer therapies and the accumulation of cardiovascular risk factors [1]. Chemotherapy, radiation therapy, and targeted treatments can all contribute to an increased risk of cardiac dysfunction, hypertension, and metabolic disorders [2]. Consequently, there is a growing interest in integrating structured exercise interventions, including the exercise-based components of cardiac rehabilitation (exCR), into the supportive care and prevention of people diagnosed with cancer [3].

Traditionally, cardiac rehabilitation (CR) is a multi‑component, guideline‑based model that combines exercise training with risk‑factor management, education, and psychosocial support [4]. In the present review, we operationalize CR specifically as its exercise‑based component, consistent with established CR prescription parameters (frequency, intensity, time, and type). Non‑exercise components (e.g. psychosocial, nutritional, risk‑factor management) are acknowledged as core elements of full CR but are not evaluated here. In oncology populations, interventions are most often delivered as exercise-led programmes rather than comprehensive CR services; focusing on the exercise component therefore aligns the review with real-world implementation and reporting [5]. This framing prevents conceptual mismatch between introduction and methods and clarifies that our analyses isolate the exercise dimension most commonly implemented and reported in oncology populations. The cardio‑oncology rehabilitation (CORE) concept provides the broader clinical context in which exCR may be integrated to mitigate therapy‑related cardiovascular risk [6–9].

Despite the growing body of evidence supporting exercise-centric approaches in oncology, access to center-based programs remains limited due to logistical barriers such as transportation, distance, and work or family responsibilities [10]. In cardiovascular medicine, home-based CR is increasingly recognised as a viable alternative or complement to centre-based delivery, supported by the broader shift toward remote monitoring and telehealth-enabled secondary prevention [11]. This evolution provides a pragmatic model for extending CR principles beyond traditional settings, particularly for populations with substantial access barriers. The COVID-19 pandemic has further exacerbated these challenges, prompting an increased interest in home-based and telehealth-supported models [12,13]. Home-based exercise programs have been proposed as an alternative to traditional, supervised centre-based programs, offering greater accessibility while maintaining similar benefits in terms of adherence, safety, and clinical outcomes [14,15].

Against this background, the effectiveness of home-based exCR aligned with CR principles in cancer care remains an active area of research. This systematic review and meta-analysis therefore evaluates adherence, safety, cardiorespiratory fitness (CRF), and health-related quality of life (HRQoL) in adults with cancer undergoing home-based exCR. In doing so, it positions exCR within cardio-oncology as a pragmatic, scalable first step toward comprehensive, multi-component CR models in this population.

## Methods

The current review adheres to the Preferred Reporting Items for Systematic Reviews and Meta-Analyses guidelines [16]. The systematic review protocol was prospectively registered in the PROSPERO database (registration number: CRD42023482624) on 24 November 2023. Study eligibility was defined according to the Population, Intervention, Comparator, and Outcome (PICO) criteria [17].

### Population

Included were randomized controlled trials (RCTs) and single-group trials enrolling adult cancer patients (aged ≥18 years), encompassing individuals preparing for treatment, currently undergoing therapy, or those who had completed their therapeutic regimens.

### Intervention

In this review, RCTs were defined as comparative studies evaluating the efficacy and safety of home-based, structured exercise programs aligned with exCR-training principles commonly applied in CR, while single-arm trials were included to assess feasibility and adherence.

CR is a multi-component, comprehensive prevention programme that may include patient assessment (including cardiac risk assessment/stratification), exercise training, education for risk-factor management, and psychosocial support. Because comprehensive CR programmes are rarely delivered and explicitly reported as complete, guideline-based services in oncology settings, we did not aim to identify ‘full CR programmes’ in cancer populations. Instead, we operationally defined eligible interventions as CR-informed (or CR-aligned) exercise components, i.e. home-delivered, structured exercise training prescribed using CR principles (FITT and intensity targets), with or without additional elements commonly embedded in CR (e.g. baseline screening, safety procedures, monitoring of symptoms/vital signs, or education related to physical activity). Therefore, we did not require that all CR core components—including formal cardiac risk stratification—be delivered or reported for inclusion. Eligible interventions were selected based on exercise-training recommendations from established CR guidance, notably the position statement from the Secondary Prevention and Rehabilitation Section of the European Association of Preventive Cardiology [18,19].

Specifically, interventions were required to include structured home-based exercise modalities, categorized as aerobic training, strength training, or combined aerobic and strength regimens. Exercise intensity criteria adhered to the principles outlined for exCR, namely moderate intensity defined as 40–69% of peak oxygen uptake (*V*O_2_ peak) or 55–74% of maximal heart rate (HR peak), and vigorous intensity at 70–85% of *V*O_2_ peak or 75–90% HR peak. Alternative validated intensity measures, including the Borg Rating of Perceived Exertion (RPE) scale (scores of 12–16) or the Talk Test, were acceptable in contexts where objective measures were impractical. Consistent with our a priori focus on the exercise domain of exCR, non-exercise CR components (e.g. psychosocial counselling, structured nutritional therapy, or formal cardiovascular risk-factor clinics) were considered out of scope unless their effects could be analytically separated from exercise.

Interventions needed to specify exercise frequency (ideally a minimum of three structured sessions per week), intensity, and duration in line with established cardiac rehabilitation standards, typically involving sessions lasting between 30–60 min. Programs meeting at least 150 min per week of moderate-intensity or 75 min per week of vigorous-intensity exercise were prioritized. Studies prescribing structured, supervised home-based exercise totaling at least 150 min per week, despite not strictly meeting frequency criteria, were also considered.

Studies assessing cardiovascular fitness outcomes, such as *V*O_2_ peak, heart rate recovery (HRR), or the 6-min walk test (6MWT), were particularly targeted for inclusion. Trials incorporating combined interventions (e.g. dietary or lifestyle modifications) were excluded unless exercise-specific effects could be isolated. Interventions could be initiated at any stage provided they met the aforementioned exercise specifications.

Throughout the manuscript, we use the terms ‘CR-informed/CR-aligned’ to avoid implying that the included interventions constituted comprehensive CR services.

### Comparator

To be eligible, studies were required to include a comparator group receiving usual care or no structured exercise intervention. In the case of trials with multiple experimental arms, inclusion was contingent upon at least one of the groups serving as a standard care or control condition, allowing for evaluation of the incremental benefit of the structured home-based exercise intervention in comparison to standard practice.

### Outcome

The outcomes of interest in this review included safety, adherence, and specific health-related outcomes relevant to exCR. Safety was evaluated through reporting and categorization of adverse events, including conditions requiring hospitalization, causing significant disability, being life-threatening, or resulting in death. Adherence was assessed as the proportion of scheduled home-based exercise sessions successfully completed by participants. Additional outcomes included CRF, muscular strength, HRQoL, and fatigue. Outcomes were systematically extracted as mean and standard deviation values from intervention and control groups. Data collected for analysis included recruitment and withdrawal percentages, adherence rates, CRF, HRQoL, fatigue scores, muscular strength measures, details of adverse events, and dropout information, documented alongside authorship details and publication years.

### Search methods

An electronic literature search was conducted in January 2025 using databases including PubMed, EMBASE, Scopus, Cochrane Library, CINAHL, and EBSCOhost, targeting studies evaluating home-based exercise interventions in the context of cardiac rehabilitation. The search encompassed publications in English available since the inception of these databases. Initial selection of relevant studies was performed based on predefined keywords. Two independent reviewers (PW & LB) screened titles and abstracts for potential inclusion. Subsequently, the full texts of potentially relevant studies were thoroughly reviewed against predefined inclusion criteria. Discrepancies between reviewers were resolved by discussion or, if necessary, by consulting a third reviewer (FD). For studies with multiple publications, the most comprehensive version containing detailed systematic data was selected. Authors were contacted directly when supplementary information was required. The complete PubMed search strategy, including all search terms, Boolean operators, field tags, and limits, is provided in Supplementary Table 3.

### Study quality and reporting

The quality and reporting of included studies were evaluated using the Tool for the assEssment of Study qualiTy and reporting in EXercise (TESTEX), a validated instrument specifically designed for exercise training studies [20]. TESTEX was selected due to its reliability and appropriateness in assessing methodological rigor and comprehensive reporting of exercise interventions. Unlike traditional quality assessment tools, TESTEX accounts for practical limitations such as participant and researcher blinding, which are particularly challenging in exercise studies. TESTEX comprises 12 criteria with varying point allocations, allowing a maximum score of 15 points. 5 points dedicated to methodological quality and 10 points to reporting clarity. Studies scoring 12 points or more were classified as ‘High Quality,’ those scoring between 7 and 11 as ‘Good Quality,’ and those scoring 6 or fewer points as ‘Low Quality’ [21].

Intervention quality was categorized based on relevance, reproducibility, methodological clarity, and accuracy in result reporting. High intervention quality indicated highly relevant, clearly described, and reproducible interventions with precise outcome reporting. Good intervention quality reflected moderately relevant interventions suitable for replication but with some reporting limitations. Low intervention quality included interventions with significant limitations in relevance, methodological clarity, reproducibility, and insufficient reporting detail.

### Data extraction

Two reviewers (PW & LB) independently assessed titles and abstracts identified in the electronic database searches. Any disagreements were resolved through consultation with a third reviewer (FD). Full-text articles deemed relevant were retrieved, and detailed data extraction was performed. Extracted data included study characteristics, participant demographics, specifics of the exercise interventions, and the reported outcomes, systematically organized in predefined data fields and tables.

### Data synthesis

For outcomes consistently reported across studies, we conducted random-effects meta-analyses using Hedges’ g as the standardized mean difference (SMD). The analysis was based on post-intervention mean values and standard deviations for intervention and control groups, as change scores or pre–post correlations were not consistently available [22].

We applied the DerSimonian–Laird method to estimate between-study variance (*τ*^2^), and statistical heterogeneity was quantified using the *I*^2^ statistic. Because only eight studies contributed to the CRF meta-analysis and seven to the HRQoL meta-analysis, subgroup analyses and meta-regression were not undertaken; potential sources of heterogeneity were therefore explored qualitatively. Formal assessment of small-study effects was also not performed because fewer than 10 studies contributed to each synthesis. Strength and fatigue outcomes were presented descriptively due to limited and inconsistent reporting. All analyses were conducted using Meta-Mar version 4.0.2.

## Results

A database and meta-search engine search were performed and identified 3510 records. After screening the titles and abstracts, it was found that 2948 publications did not meet the inclusion criteria. Of the 142 full-text publications, 133 records were excluded. Finally, nine publications met the inclusion criteria for this systematic review and meta-analysis [23–31]. Figure 1 provides an overview of the study flow process.

**Figure 1.**
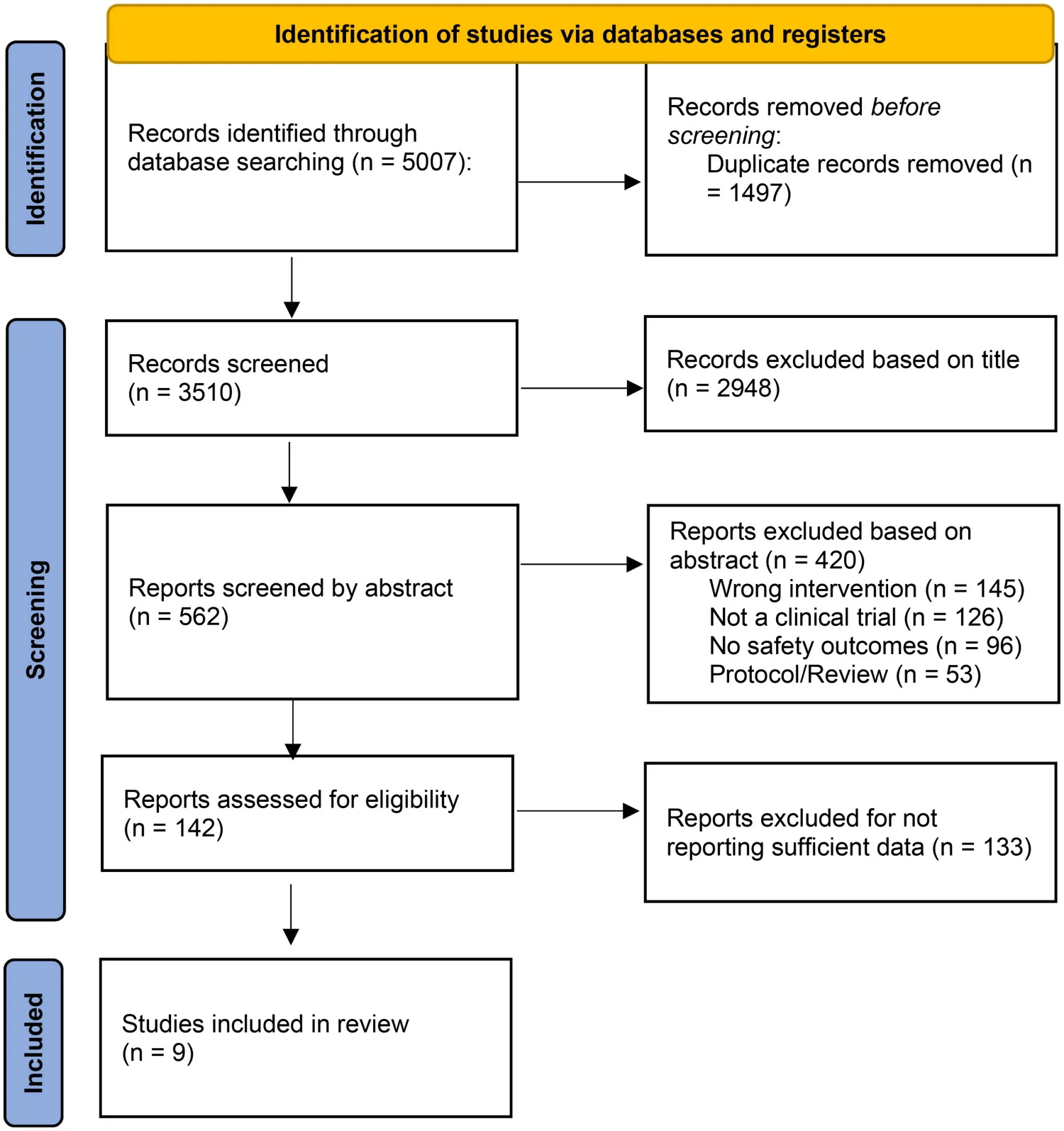
PRISMA flowchart of the selection process.

### Characteristics of participants

The characteristics of all nine included studies and their participants are shown in Table 1 The methodological characteristics of studies and adherence and safety outcomes within the studies are presented in Tables 2 and 3. Particularly all included studies were of RCT design and were conducted in USA [23,30], France [24], China [25], Germany [26], Spain [27], Netherlands [28], Australia [29], and Canada [31].

**Table 1.** Characteristics of studies that evaluated the effects of home-based exercise.

Study	Treatment	Cancer	Design	*N*	Sex	Mean age	Duration (weeks)
Portela [30]^a^	Post-surgery, post or without adjuvant therapy	BC	Pilot feasibility RCT (1:1:1)	34	W	52.9	13
Santa Mina [31]^b^	During ADT	PC	RCT (1:1)	66	M	72.1	12
Cornette [24]	During adjuvant CT or RT	BC	RCT (1:1)	42	W	52	27
Galiano-Castillo [27]	Post adjuvant therapy except hormone treatment	BC	RCT (1:1)	81	W	48.3	8
Gehring [28]	Post treatment	Gliomas	Pilot RCT (2:1)	34	W/M	48	24
Dong [25]	Post-surgery, post CT or RT	BC	RCT (1:1)	60	W	49.8	12
Falz [26]	Post-surgery	BC, PC and CC	RCT (1:1)	148	W/M	54	12
Blarigan [23]	Never treated, active surveillance	PC	RCT (1:1)	51	M	62.9	16
Granger [29]	Post-surgery	LC	RCT (1:1)	116	W/M	66.4	12

BC = breast cancer, ADT = androgen deprivation therapy, PC = prostate cancer, CC = colorectal cancer, LC = lung cancer.

^a^Gym-exercise, home-exercise and standard care group.

^b^Aerobic exercise and resistance exercise training group.

**Table 2.** Characteristics of home-based exercise programs.

Study	Exercise	Type^a^	Intensity^a^	Frequency (sessions per week)^a^	Duration, sets, repetitions^a^	Gradual adjustment^a^	Monitoring, feedback
Portela [30]	AE and RE	Walking/elastic bands	RPE 12–16/ RPE 13–15	3/2	30 min/2–3 sets, 10–15 repetitions	NR/repetitions and thickness of bands	1 phone call/week, 1 personal meeting/ month
Santa Mina [31]^b^	AE and RE	Walking or other AE/elastic bands	60–80% HRR/RPE 12–15, 60–80% 1-RM	3–5/3–5	30–60 min/30–60 min, 2–3 sets, 10–12 repetitions	Duration and training HR/ thickness of bands	Heart rate monitor, log book, 1 phone call/2 weeks
Cornette [24]	AE and RE	Cycle ergometer or walking/elastic bands	Power or HR determinated by 1-VT	2/1	20–40 min/2 sets, 8–12 repetitions	Duration/NR	Diary, heart rate monitor, 1 phone call/week
Galiano-Castillo [27]	AE and RE	Battery of specific exercises	ACSM guidelines	3 AE and RE combined	90 min AE and RE combined	NR/NR	Each session online with messaging, 3 message or video session/week, phone call option
Gehring [28]	AE	Any type of AE	60–85% HRmax	3	NR	NR	Heart rate monitor, online platform, 1 feedback e-mail/week, phone call option
Dong [25]	AE and RE	Remote exercises	RPE 13-16/70-80% 1-RM	4/3	NR/30 min	Yes, but not specified	Each session online
Falz [26]	Strength–endurance training	Body weight exercises	75% HRpeak, 5–8 CR10 scale	2–3 or more	30 min, 4 sets, 40 s loading and 20 s recovery	Intensity	Each session online, heart rate monitor, online platform
Blarigan [23]	AE	Walking	45–80% *V*O_2_ peak	3–4	20–60 min	Frequency, duration, intensity	Heart rate monitor, online platform, 1 phone call/week
Granger [29]	AE and RE	Battery of specific exercises	NR/NR	NR/NR	NR/NR	NR	1 phone call/week, diary, physical activity monitor (number of steps)

AE = aerobic exercise, RE = resistance exercise, RPE = Borg scale of perceived exertion, NR = not reported, HRR = heart rate reserve, 1-RM = one repetition maximum, HR = heart rate, 1-VT = first ventilatory threshold, ACSM = the American College of Sports Medicine, HRmax = maximum heart rate, CR10 scale – Category-ratio scale, *V*O_2_ peak = peak oxygen uptake.

^a^Aerobic exercise/resistance exercise.

**Table 3.** Completion, adherence, adverse events and dropouts.

Study	Follow-up (weeks)	Completion IG/CG (%)	Dropouts IG/CG (*n*)	Exercise adherence (%)	Reported adverse events IG/CG (*n*)
Portela [30]^a^	13	65.5/100	10/0	78.5	NR
Santa Mina [31]^b^	12	87.5/85.3	4/5	NR	No adverse events
Cornette [24]	27	86.4/72.7	5/7	88	No adverse events/NR
Galiano-Castillo [27]	8	97.5/90.2	4/5	NR	NR
Gehring [28]	24	82.6/81.8	4/2	79	No adverse events/NR
Dong [25]	12	86.7/80	4/6	NR	No adverse events/NR
Falz [26]	12	81.5/83.3	14/12	56.4	10/6
Blarigan [23]	16	92.3/92	4/2	94	51/45
Granger [29]	12	81/96.6	11/2	79.3	1/0

Adverse-event counts are reproduced as reported in the original studies and may include all-cause events; definitions, severity grading, and attribution to exercise were inconsistent.

IG = intervention group, CG = control group, NR = not reported.

^a^Gym-exercise, home-exercise and standard care group.

^b^Aerobic exercise and resistance exercise training group.

Participants included in the reviewed studies were cancer survivors or patients in various stages of treatment. The type of cancer and the timing of the intervention varied substantially across studies. Breast cancer was the most frequently studied diagnosis, present in 5 out of 9 studies [24–27,30]. Prostate cancer was examined in 3 studies [23,26,31], and colorectal cancer in 1 study, as part of a combined cohort [26]. Gliomas were addressed in 1 study [28], and lung cancer in another [29].

Most participants were in the post-treatment phase or following surgery, though some interventions were conducted during adjuvant therapy [24,31], or during active surveillance/hormone therapy [23,31]. The studies by Falz et al. [26] and Portela et al. also included mixed or combined groups [30]. Sample sizes ranged from 34 to 148 participants. The mean age of participants varied between 48.0 and 72.1 years, with an average across studies in the early fifties. The youngest population was observed in Galiano-Castillo et al. (48.3 years), and the oldest in Santa Mina et al. (72.1 years) [27,31].

In terms of gender, most studies focused on single-gender populations, reflecting the nature of the cancers studied. However, four studies included both women and men [26,28–30].

### Characteristics of interventions

The characteristics of home-based exercise interventions are summarized in Table 2. The intervention duration ranged from 8 to 27 weeks, reflecting variability in study aims and cancer populations. All studies implemented aerobic exercise (AE) components, and seven studies combined AE with resistance exercise (RE) [24–28,30,31]. Only two studies focused on aerobic training exclusively [23,28].

### Exercise modality

Walking was the most common aerobic modality [23,30,31], with other methods including cycle ergometers [24] and general aerobic movements [28]. Resistance training was primarily conducted using elastic bands [24,25,30,31] or bodyweight exercises [26]. Galiano-Castillo et al. [27] and Granger et al. [29] implemented structured batteries of exercises.

Exercise frequency typically ranged from 2 to 5 sessions per week for AE and 1 to 3 sessions per week for RE. Most studies prescribed 3 sessions weekly for aerobic training [24,25,27,28,30], with the highest being 5 sessions/week in Santa Mina et al. [31]. Session durations varied from 20 to 90 min, depending on whether AE and RE were combined.

### Intensity and progression

Exercise intensity was most commonly prescribed at a moderate to vigorous level, determined using heart rate targets, Borg’s RPE scale [12–16], or thresholds [24–26,28,30,31]. Several studies applied progressive individualization of load based on patient capacity and adaptation [23,24,26,30,31], although some did not report specific progression methods [25,27–29].

### Monitoring and feedback

All studies implemented some form of monitoring and feedback, a key feature of home-based cardiac rehabilitation. Heart rate monitoring was used in six studies [23,24,26,28,30,31]. Telecommunication support included phone calls, online platforms, messaging systems, or a combination. Supervised real-time contact was used in two studies with interactive feedback during sessions [26,27], while others relied on asynchronous post-exercise feedback [23,24,28,30,31]. Feedback was typically delivered weekly, most often *via* phone calls or email messaging, and occasionally included personal meetings [30]. The professionals delivering exercise support ranged from physiotherapists and exercise physiologists to research staff, although not all studies specified the background of the providers.

### Completion rate and adherence

Adherence to the home-based exercise interventions was reported in most studies, though the methodology for its assessment varied. Exercise adherence was explicitly quantified in five studies [24,26,28–30], with reported adherence rates ranging from 56.4% to 94%. The highest adherence was observed in Blarigan et al. (94%) and Cornette et al. (88%) [23,24]. In contrast, four studies [25,27,30,31] did not report adherence rates or the method of adherence assessment, even though retention was often high. For instance, Galiano-Castillo et al. [27] reported 97.5% completion in the intervention group despite lacking explicit adherence data.

Completion rates for intervention groups ranged from 65.5% to 97.5% [27,30]. The majority of studies reported completion rates over 80% in the intervention group [23–26,28,29,31]. Dropout rates were generally low and comparable between intervention and control groups. The highest number of dropouts in the intervention group was reported by Falz et al. (*n* = 14) and Granger et al. (*n* = 11), possibly due to the duration and complexity of the program [26,29]. Most other studies reported ≤5 dropouts in the intervention arms.

### Adverse events

Adverse events were inconsistently and incompletely reported across the included studies. Several trials reported no adverse events or only isolated minor events, whereas other studies reported event counts without sufficient information to determine severity, exercise relatedness, or whether all-cause or exercise-related events were being captured. Some trials did not report safety outcomes. Definitions, severity grading, attribution, and denominators were therefore not standardized. Accordingly, no consistent excess of serious exercise-related harm was apparent, but the available evidence is insufficient to establish the safety of home-based exCR conclusively.

### Outcomes

#### Assessment of outcomes

An overview of the methods and instruments used to evaluate health-related outcomes across the nine included studies is summarized in Supplementary Table 1. All studies (9/9, 100%) assessed CRF. The most frequently used method was *V*O_2_ peak or *V*O_2_ max, employed in 6 out of 9 studies (66%) [23–27,30]. Field tests were also applied: 6-min walk test in 2 studies (22%) [28,31], and 12-minute walk test in 1 study (11%) [30]. It is important to acknowledge that the methods used to assess CRF varied across studies, with some employing direct CPET to determine *V*O_2_ peak or *V*O_2_ max, while others relied on submaximal field tests such as the six-minute walk test. These methodological differences may influence the precision and comparability of fitness estimates, potentially contributing to observed heterogeneity.

HRQoL was evaluated in 8 studies (88%) [23–28,30,31], with the most common tool being the EORTC QLQ-C30 used in 3 studies (38%) [24,27,29]. Fatigue was assessed in 4 studies (45%) using a variety of tools: FACT-F, Piper Fatigue Scale, and Fatigue Severity Scale, each in one study (25% respectively). One study [27] listed fatigue among outcomes but did not specify the assessment method.

Muscle strength was measured in 6 studies (66%) [24–27,29,30], with the handgrip test being the most frequent method (4/6, 66%). Additional methods included quadriceps dynamometry, sit-to-stand test, arm lifting, and isometric trunk strength. Anxiety and depression were less frequently assessed. Anxiety was measured in 3 studies (33%) using the Hospital Anxiety and Depression Scale (HADS), the MAX-PC, and the S-Anxiety inventory. Depression was evaluated in 2 studies (22%) [25,28], both using HADS and DASS scales.

Additional outcomes included self-reported physical activity assessed by IPAQ, pain (Brief Pain Inventory), sleep, and physical function. The diversity of assessment tools reflects the multidimensional focus of home-based exercise interventions targeting physical, psychological, and functional domains in cancer populations.

### Meta-analysis outcomes

#### Cardiorespiratory fitness

CRF was assessed in eight studies using *V*O_2_ peak, *V*O_2_ max, or walk-based field tests. Meta-analysis of these studies demonstrated a moderate pooled effect size in favor of the intervention group, with a standardized mean difference (SMD) of 0.42 [95% CI: 0.09 to 0.75, *I*^2^ = 62%], indicating improved cardiorespiratory capacity as a result of home-based exCR. The forest plot is shown in Figure 2A.

**Figure 2.**
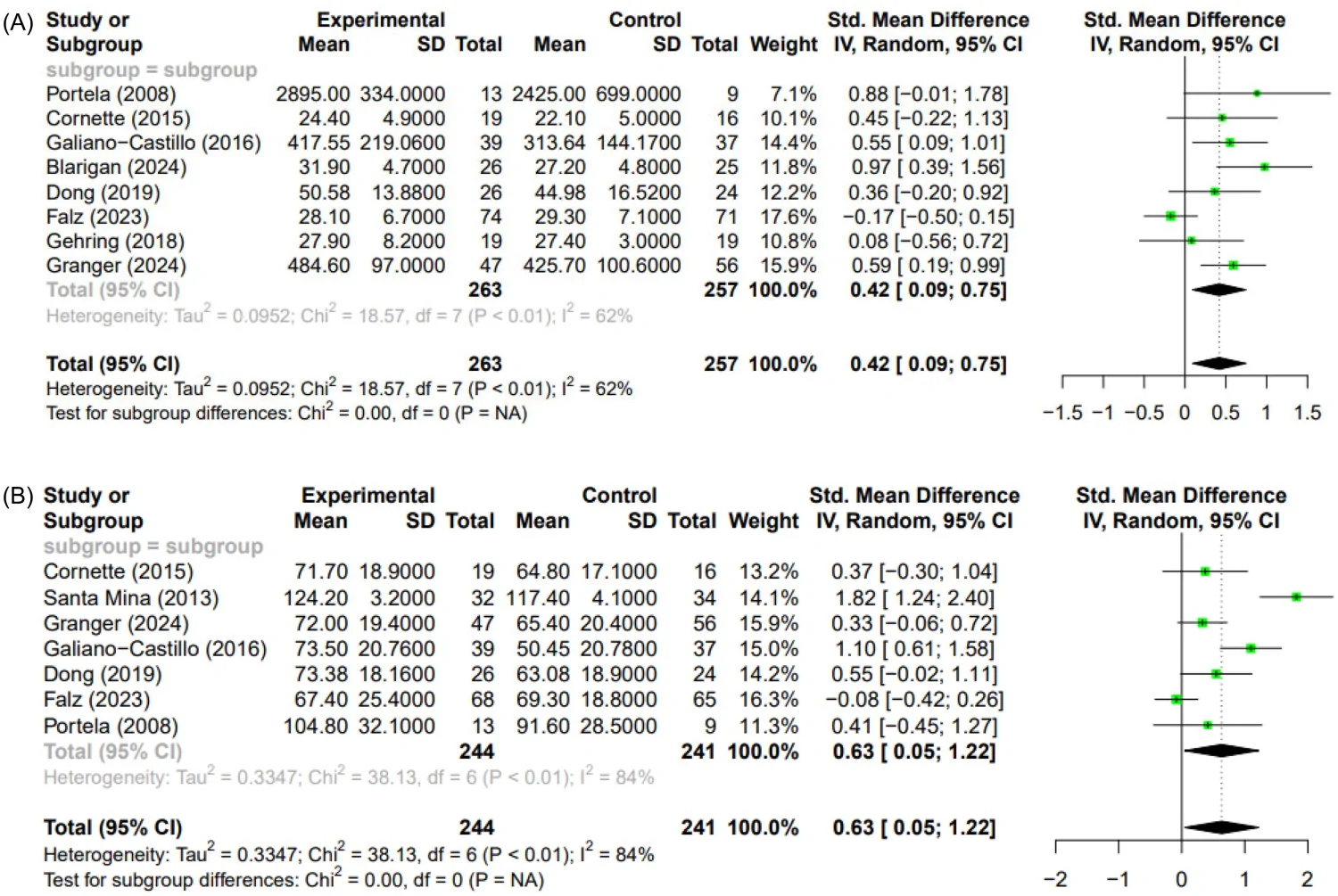
A. Forest plot of meta-analysis for CRF outcomes. B. Forest plot of meta-analysis for health-related HRQoL.

#### Health-related quality of life

HRQoL was reported in seven studies using validated patient-reported outcome measures, including the EORTC QLQ-C30, FACT-B, SF-36, and others. Meta-analysis showed a moderate pooled effect size in favor of the intervention group (SMD = 0.63 [95% CI: 0.05–1.22]), although substantial heterogeneity was detected (*I*^2^ = 84%), suggesting variability in measurement tools and populations. Notably, the study by Falz et al. [26] did not report significant improvements in HRQoL, possibly due to relatively high baseline quality-of-life scores, leaving less room for measurable improvement, and the intervention intensity or modality potentially not being sufficient to induce noticeable changes in patient-reported outcomes. The forest plot is provided in Figure 2B.

#### Strength and fatigue

Muscle strength was assessed in five studies [24,25,29–31]. Various objective methods were used, most commonly handgrip strength or composite functional tests. While within-group improvements in strength were observed across several intervention groups, only Dong et al. [25] reported a statistically significant between-group difference in favor of the intervention group (*p* = 0.02). Overall, the findings did not consistently favor home-based exercise over control conditions.

Fatigue was evaluated in three studies [24,29,31], each using different measurement instruments. Although minor within-group improvements were seen in some intervention arms, none of the studies demonstrated a significant difference between intervention and control groups. Detailed descriptive results for strength and fatigue are presented in Supplementary Table 2.

#### Study reporting and quality

The quality of the included studies was assessed using the TESTEX scale (Table 4). The mean total score was 10.0/15 (range 7–13), indicating generally good-to-high methodological and reporting quality. Based on the prespecified thresholds, seven studies were classified as good quality (7–11 points) and two as high quality (≥12 points); none was classified as low quality (≤6 points). The methodological-quality component averaged 3.7/5 (range 2–5), with the highest scores in Gehring et al. [28] and Granger et al. [29]. The reporting component averaged 6.3/10 (range 3–10), with the highest score in Blarigan et al. [23] and the lowest in Dong et al. [25].

**Table 4.** Study quality using TESTEX scale.

Study	Study quality (max 5)	Study reporting (max 10)	Total score (max 15)
Portela (2008)	3	7	10
Santa Mina (2013)	4	8	12
Cornette (2015)	2	6	8
Galiano-Castillo (2016)	4	5	9
Gehring (2018)	5	4	9
Dong (2019)	4	3	7
Falz (2023)	3	8	11
Blarigan (2024)	3	10	13
Granger (2024)	5	6	11

TESTEX = Tool for the Assessment of Study Quality and Reporting in Exercise.

## Discussion

This systematic review and meta-analysis found that home-based exCR was associated with moderate improvements in CRF and HRQoL among people diagnosed with cancer. These pooled effects support potential benefit but should be interpreted alongside the moderate-to-substantial heterogeneity and the limited number of trials. The findings are broadly consistent with reports on center-based exercise training within CR services and with international support for home-based and hybrid exCR delivery models [32–34]. Importantly, this review isolates the exercise-based domain of CR as currently implemented in oncology; future trials should evaluate fully integrated, multi-component CORE models that combine supervised or telemonitored exercise with cardiovascular risk management, psychosocial and nutritional support, and long-term follow-up [33,35].

The observed heterogeneity should be considered when interpreting the pooled estimates. For CRF (*I*^2^ = 62%), plausible sources include the use of direct cardiopulmonary exercise testing versus field-based walk tests, differences in cancer diagnosis and treatment phase, baseline fitness, intervention duration (8–27 weeks), exercise mode and dose, monitoring intensity, and adherence. For HRQoL (*I*^2^ = 84%), heterogeneity may additionally reflect the use of conceptually different instruments (including the EORTC QLQ-C30, FACT measures, SF-12/SF-36, and EPIC-26), variation in baseline HRQoL and possible ceiling effects, and differences in the domains captured by each scale. With only eight CRF studies and seven HRQoL studies, formal subgroup analyses or meta-regression would have been underpowered and were not undertaken. A random-effects model allows for between-study variability but does not resolve this clinical and methodological heterogeneity; the pooled SMDs should therefore be interpreted as average effects across diverse settings rather than effects expected in every cancer population or intervention format. The direction of effect supports potential benefit, but the magnitude and applicability of that benefit remain uncertain.

Recent research in the field of CORE underscore the gap between existing guidelines and practical implementation of home-based supportive models [36–41]. Despite strong recommendations for integrated exercise approaches covering physical activity, nutrition, and psychosocial care, only a minority of interventions address all six cardinal domains [40]. This implementation gap persists despite the increasing call to action from cardio-oncology leaders to adopt CORE programs that integrate cardiovascular risk management, supervised or telemonitored exercise, nutritional counseling, and long-term support. As highlighted by Díaz-Balboa et al. [42], these programs mirror traditional exCR but are tailored to the unique needs of oncology populations through structured three-phase models and interdisciplinary collaboration. Our findings confirm the feasibility of deploying core exercise components *via* home-based delivery, even though comprehensive integration remains limited.

The present findings are also consistent with evidence that technology-supported remote exercise delivery can achieve high adherence. Adherence across the included studies was generally high, with most reporting trials exceeding 75%, suggesting good feasibility. This is in line with Su et al. (2025), who reported a pooled adherence rate of 76.2% across more than 10,000 participants in home-based cancer exercise trials [43]. The adherence rates observed in this review also appear comparable to those reported for center-based CR programs. For example, Singh et al. (2021) reported adherence approaching 82% in supervised, center-based exercise interventions in breast cancer survivors, indicating that home-based models may support engagement when designed with adequate monitoring, support, and patient education [44]. These findings underscore the potential of home-based exCR as a flexible alternative or complement to center-based programs, particularly when logistical or health-related barriers limit access [34]. Notably, two of the nine included studies specifically targeted older adults (≥65 years), supporting the relevance of home-based models in geriatric oncology [45].

In addition to adherence, dropout and withdrawal rates provide important insight into the acceptability and sustainability of home-based exCR. Across the nine trials included in this review, we observed a total dropout rate of 15.2%, with 60 participants withdrawing from intervention groups and 41 from control arms. These figures are moderately higher than the pooled dropout rate of 9.6% reported in a recent analysis of exercise oncology trials, where predictors such as higher BMI, lower education level, and certain exercise modalities were significantly associated with greater attrition [46]. While our findings remain within an acceptable range, future home-based exCR programs should consider tailored support strategies to minimize attrition among vulnerable subgroups.

The available safety findings should be interpreted cautiously. Although no consistent excess of serious exercise-related events was evident, adverse-event definitions, ascertainment, severity grading, and attribution were inconsistent, and several studies provided no detailed safety data. The low number of reported serious events may therefore reflect under-ascertainment or underreporting rather than definitive evidence of safety. Future trials should prospectively define adverse events, report denominators and event attribution by group, and use standardized harms-reporting frameworks [47–50].

Our findings showed inconsistent results for muscular strength and fatigue, which were typically reported descriptively and assessed using heterogeneous tools. Only Dong et al. [25] reported a statistically significant improvement in strength. This echoes prior observations in telehealth-based cancer rehabilitation, where aerobic components are more robustly implemented and measured than resistance training [43]. Despite the clinical relevance of strength and fatigue as outcomes in cancer rehabilitation, their underreporting limits broader conclusions. This is especially notable given that resistance training is a core recommendation in home-based exCR protocols [51]. Current guidelines emphasize incorporating both aerobic and resistance modalities for comprehensive benefit, particularly for preserving or restoring muscle mass and function in oncology populations [52].

Previous studies confirm that resistance training leads to significant improvements in muscle strength and physical functioning among cancer survivors [53–55]. Yet, our review suggests that resistance components were either underprescribed, insufficiently progressed, or inadequately monitored and reported. This underrepresentation calls for greater standardization in strength assessment and clearer prescription reporting in future trials. Moreover, the limited strength outcomes may reflect a broader issue in implementation while aerobic prescriptions are more frequently and consistently applied in home-based settings, resistance training remains challenging to deliver remotely, despite its documented safety and feasibility.

Standardized strength assessments and targeted resistance prescriptions are needed. Despite encouraging feasibility findings, broad implementation of CORE programs in routine oncology care remains limited. As emphasized, successful scaling will require institutional support, integration with primary care, and professional coordination among oncologists, cardiologists, and rehabilitation specialists [42].

## Limitations

This review has several limitations that should be considered when interpreting the findings. First, the evidence base was derived from a small number of studies with relatively small sample sizes, which reduces statistical power, increases uncertainty around pooled estimates, and limits the ability to explore clinically meaningful subgroups. Second, there was substantial clinical and methodological heterogeneity across trials, including differences in cancer diagnoses and treatment phases, intervention duration and progression, exercise modality and dose, supervision/monitoring approaches, and outcome measurement instruments potentially contributing to variability in effect estimates.

Third, reporting of safety procedures and cardiovascular risk assessment/risk stratification was inconsistent. Incomplete and non-standardized adverse-event definitions, severity grading, attribution, and denominators preclude firm conclusions regarding safety across settings and risk profiles; low reported event counts should not be interpreted as evidence of absence of harm. Fourth, the generalisability of findings may be constrained by selective recruitment and underrepresentation of certain groups, as well as by the controlled conditions of trials relative to routine care. In addition, given the small number of studies available for several outcomes, publication bias and small-study effects cannot be ruled out.

Finally, our operational focus was on the exercise-training component aligned with exCR principles rather than comprehensive, multi-component CR programmes. Therefore, the results should not be interpreted as evidence for fully integrated CR/CORE service delivery in oncology populations, and future trials should evaluate multi-component models that incorporate formal cardiovascular risk management alongside structured exercise.

## Conclusion

In conclusion, home-based exCR is a feasible strategy for delivering exercise rehabilitation in oncology and may improve CRF and HRQoL. It may serve as an accessible alternative or complement to center-based rehabilitation. However, clinical and methodological heterogeneity and incomplete adverse-event reporting limit certainty, particularly regarding the magnitude of benefit and safety. Future trials should include longer-term follow-up, standardized outcome measures, and systematic reporting of adverse events and implementation outcomes.

## Supplementary Material

Supplemental Material

Supplementary_Table_3_PubMed_search_strategy.docx

Supplementary_Table_2_revised.docx

Supplementary_Table_1_Outcomes.docx

## Data Availability

All data generated or analysed during this study are included in this published article and its supplementary information files. Additional datasets are available from the corresponding author on reasonable request.

## Open scholarship

This article has earned the Center for Open Science badges for Preregistered. The materials are openly accessible at PROSPERO: CRD42023482624.
