# Supplementary material for: Home-based exercise interventions incorporating cardiac rehabilitation principles for people with cancer: a systematic review and meta-analysis
Source: Ann Med. 2026 Sep 29;58(1):2726615. doi: 10.1080/07853890.2026.2726615 (PMC13629760; doi:10.1080/07853890.2026.2726615)
Supplement: Supplementary file 1 [file IANN_A_2726615_SM5520.docx]

**Supplementary table 1** Characteristics of outcomes in home-based exercise studies

| **Study** | **Primary/Secondary Outcomes** | **Outcomes** | **Instrument/Method and number of studies**  (n = 9) |
| --- | --- | --- | --- |
| Portela (2008) | Functional evaluation, shoulder range of motion, CRF, handgrip strength, BMI, HRQL | Cardiorespiratory fitness  (n = 9, 100%) | VO2peak or VO2max, n = 6 (66%)  6-min walk test, n = 2 (22%)  12-min walk test, n = 1 (11%) |
| Santa Mina (2013) | Fatigue, HRQL/adherence, PA volume, CRF | Health-related Quality of life (n = 8, 88%) | EORTC QLQ-C30, n = 3 (38%)  SF-36, n = 2 (25%)  SF-12, n = 1 (13%)  EPIC-26, n = 1 (13%)  FACT-B, n = 1 (13%)  FACT-P, n = 1 (13%) |
| Cornette (2015) | CRF/strength, fatigue, HRQL, PA level, anxiety, depression | Strength  (n = 6, 66%) | Handgrip test, n = 4 (66%)  Quadriceps dynamometry, n = 2 (33%)  Sit-to-stand test, n = 2 (33%)  Arm lifting test, n = 1 (17%)  Abdominal/back isometry, n =1 (17%) |
| Galiano-Castillo (2016) | HRQL, pain, strength, fatigue/CRF, cognition | Fatigue  (n = 4, 45%) | FACT-F, n = 1 (25%)  The Piper Fatigue Scale, n = 1 (25%)  The Fatigue Severity Scale, n = 1 (25%)  Only mentioned in outcomes, n = 1 (33%) |
| Gehring (2018) | Self-reported PA, CRF, cognition, | Anxiety  (n = 3, 33%) | Depression Anxiety Stress Scale, n = 1 (33%)  HADS, n = 1 (33%)  MAX-PC, n = 1 (33%)  S-Anxiety, n = 1 (33%) |
| Dong (2019) | HRQL/strength, CRF | Depression  (n = 2, 22%) | Depression Anxiety Stress Scale, n = 1 (50%)  HADS, n = 1 (50%) |
| Falz (2023) | CRF/cardiopulmonary exercise testing parameters, anthropometry, body cell mass, C-reactive protein, HRQL | Sleep  (n = 1, 11%) | Patient- reported/not specified, n = 1 (100%) |
| Blarigan (2024) | CRF/ansthropometry, anxiety, HRQL | Pain  (n = 1, 11%) | The Brief Pain Inventory, n = 1 (100%) |
| Granger (2024) | Self-reported physical function/objectively measured physical function, CRF, strength, PA level, fatigue, sleep, distress, financial toxicity, HRQL | Self-reported PA  (n = 3, 33%) | IPAQ, n = 3 (100%) |
|  |  | Physical function  (n = 2, 22%) | EORTC QLQ-C30, n = 1 (50%)  Short Physical Performance Battery, n = 1 (50%)  DASH, n = 1 (50%) |

CRF = cardiorespiratory fitness, BMI = body mass index, HRQL = Health-related Quality of life, VO2peak = peak oxygen uptake, VO2max = maximum oxygen uptake, PA = physical activity, EORTC QLQ-C30 = the European Organization for Research and Treatment of Cancer Quality of Life Questionnaire, SF-36 = 36-item Short Form Health Survey, SF-12 = 12-item Short Form Health Survey, EPIC-26 = Expanded Prostate Cancer Index Composite, FACT-B = the Functional Assessment of Cancer Therapy-Breast, FACT-P = the Functional Assessment of Cancer Therapy-Prostate, FACT-F = the Functional Assessment of Cancer Therapy-Fatigue, MAX-PC = the Memorial Anxiety Scale for Prostate Cancer, State Anxiety Inventory for Adults, HADS = the Hospital Anxiety and Depression Scale, the International Physical Activity Questionnaire = IPAQ, DASH = the Disabilities of the Arm, Shoulder and Hand questionaire
