# Supplementary material for: Home-based exercise interventions incorporating cardiac rehabilitation principles for people with cancer: a systematic review and meta-analysis
Source: Ann Med. 2026 Sep 29;58(1):2726615. doi: 10.1080/07853890.2026.2726615 (PMC13629760; doi:10.1080/07853890.2026.2726615)
Supplement: Supplementary file 2 [file IANN_A_2726615_SM5521.docx]

Supplementary Table 2. Strength and fatigue outcomes

| Study | Experimental group Baseline / Post-intervention | Control group Baseline / Post-intervention | Between-group significance | Outcome domain |
| --- | --- | --- | --- | --- |
| Portela (30) | 41.2 (16.6) / 40 (11.9) | 37.8 (13.5) / 34.4 (11.3) | NR | Strength |
| Cornette (24) | 25.3 (8.7) / 24 (5.5) | 28.7 (8.4) / 28.2 (8.3) | 0.15 | Strength |
| Dong (25) | 14.8 (2.9) / 19.3 (4.3)* | 15.2 (3.4) / 15.7 (3.7) | 0.02* | Strength |
| Santa Mina (31) | 63.9 (2.6) / 69.6 (2.0) | 64.5 (2.7) / 68.9 (2.3) | 0.79 | Strength |
| Granger (29) | 30.9 (10.0) / 31.9 (9.7) | 29.0 (8.7) / 27.6 (9.7) | NR | Strength |
| Cornette (24) | 48.8 (20.1) / 41.9 (14.8) | 54.4 (19.4) / 49.8 (18.4) | 0.11 | Fatigue |
| Santa Mina (31) | 42.0 (1.5) / 42.2 (1.3) | 38.1 (2.1) / 35.6 (2.2) | 0.62 | Fatigue |
| Granger (29) | 3.0 (2.4) / 3.2 (2.6) | 3.3 (2.5) / 3.8 (2.7) | NR | Fatigue |

Values are expressed as mean (Standard deviation)

*statistical significance p < 0.05
