# Supplementary material for: Home-based exercise interventions incorporating cardiac rehabilitation principles for people with cancer: a systematic review and meta-analysis
Source: Ann Med. 2026 Sep 29;58(1):2726615. doi: 10.1080/07853890.2026.2726615 (PMC13629760; doi:10.1080/07853890.2026.2726615)
Supplement: Supplementary file 3 [file IANN_A_2726615_SM5522.docx]

**Supplementary Table 3. Full electronic search strategy for PubMed**

| **Item** | **Details** |
| --- | --- |
| **Database/platform** | PubMed/MEDLINE |
| **Date searched** | January 2025 |
| **Coverage** | Database inception to January 2025 |
| **Limits** | English language; no other filters applied |
| **Search line 1: cancer concept** | "Neoplasms"[MeSH Terms] OR cancer[Title/Abstract] |
| **Search line 2: exercise concept** | "Exercise"[MeSH Terms] OR exercis*[Title/Abstract] OR (physical[Title/Abstract] AND activit*[Title/Abstract]) OR (resistance[Title/Abstract] AND training[Title/Abstract]) OR aerobic[Title/Abstract] OR sport*[Title/Abstract] OR walk*[Title/Abstract] OR move*[Title/Abstract] OR active[Title/Abstract] OR lifestyle*[Title/Abstract] OR performance[Title/Abstract] OR (training[Title/Abstract] AND intervention*[Title/Abstract]) OR (sedentary[Title/Abstract] AND behavio*[Title/Abstract]) |
| **Final combination** | #1 AND #2 |
| **Full reproducible query** | ("Neoplasms"[MeSH Terms] OR cancer[Title/Abstract]) AND ("Exercise"[MeSH Terms] OR exercis*[Title/Abstract] OR (physical[Title/Abstract] AND activit*[Title/Abstract]) OR (resistance[Title/Abstract] AND training[Title/Abstract]) OR aerobic[Title/Abstract] OR sport*[Title/Abstract] OR walk*[Title/Abstract] OR move*[Title/Abstract] OR active[Title/Abstract] OR lifestyle*[Title/Abstract] OR performance[Title/Abstract] OR (training[Title/Abstract] AND intervention*[Title/Abstract]) OR (sedentary[Title/Abstract] AND behavio*[Title/Abstract])) |

*Note: The strategy is presented with explicit PubMed field tags and Boolean grouping to ensure reproducibility.*
